# Supplementary material for: DENcode: A model for haplotype-informed transmission probability of dengue virus
Source: PLoS Comput Biol. 2026 May 20;22(5):e1014316. doi: 10.1371/journal.pcbi.1014316 (PMC13211310; doi:10.1371/journal.pcbi.1014316)
Supplement: S1 Fig — Created in BioRender. Stone, H. (2026) https://BioRender.com/urvslx0. (DOCX) [file pcbi.1014316.s001.docx]

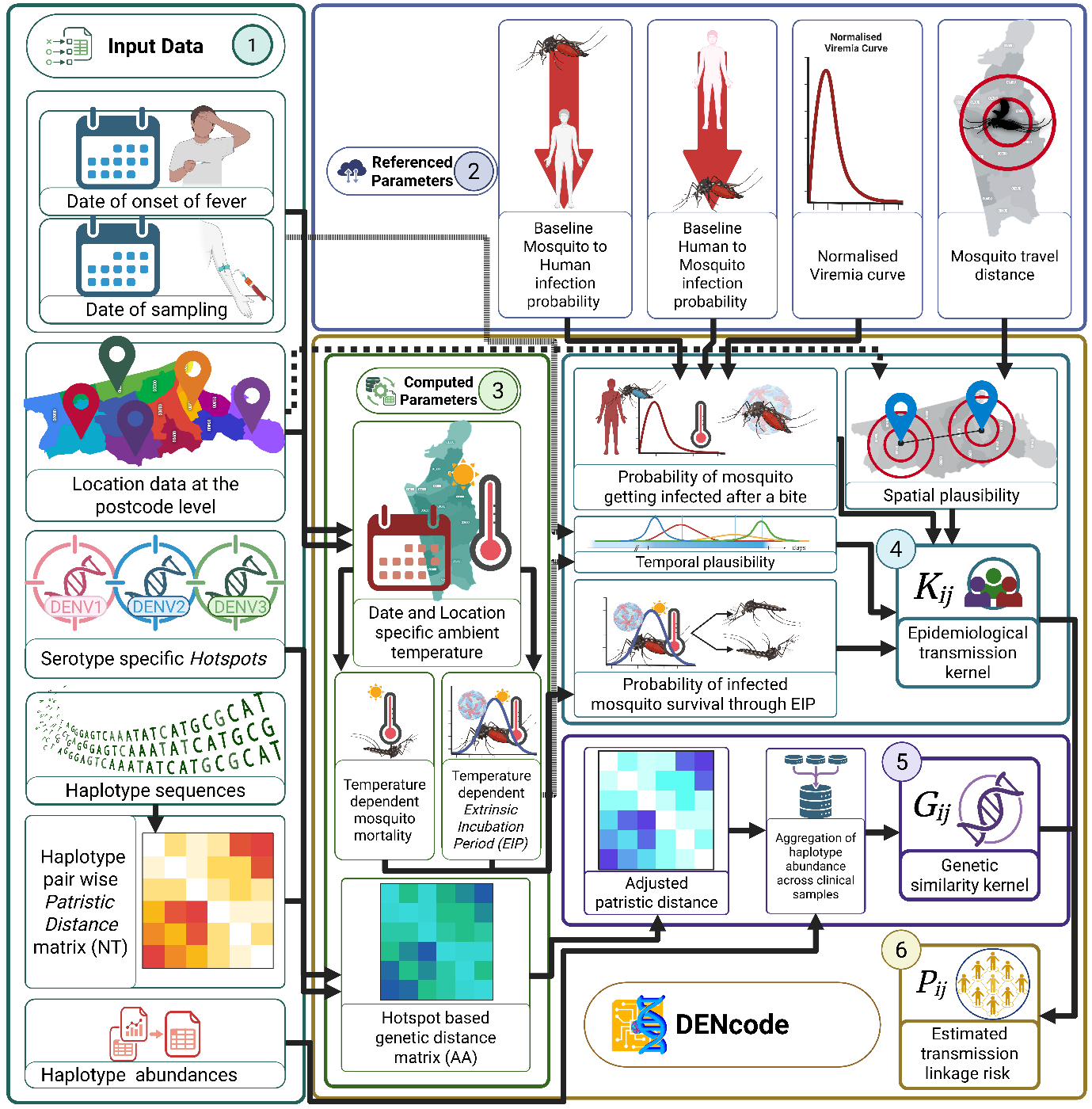


S1 Fig. Process flow diagram of DENcode.  Input data (1) include epidemiological dates, geocoded locations, serotype-specific hotspot data, and within-host haplotype sequences. Referenced parameters (2) define baseline transmission probabilities and biological constraints. Computed parameters (3) integrate temperature-dependent vector dynamics and spatial genetic structure. The epidemiological kernel $\boldsymbol{K}_{\boldsymbol{ij}}$ (4) combines spatial plausibility, temporal compatibility, and extrinsic incubation period survival probability. The genetic kernel $\boldsymbol{G}_{\boldsymbol{ij}}$ (5) aggregates haplotype-level genetic distances. Finally, DENcode (6) estimates pairwise transmission probability $\boldsymbol{P}_{\boldsymbol{ij}}$by integrating epidemiological and genetic kernels. Framework uses complete haplotype sequences to capture within-host diversity, enabling high-resolution outbreak investigation. Created in BioRender. Stone, H. (2026) https://BioRender.com/urvslx0
