# Supplementary material for: DENcode: A model for haplotype-informed transmission probability of dengue virus
Source: PLoS Comput Biol. 2026 May 20;22(5):e1014316. doi: 10.1371/journal.pcbi.1014316 (PMC13211310; doi:10.1371/journal.pcbi.1014316)
Supplement: S3 Fig — (DOCX) [file pcbi.1014316.s003.docx]

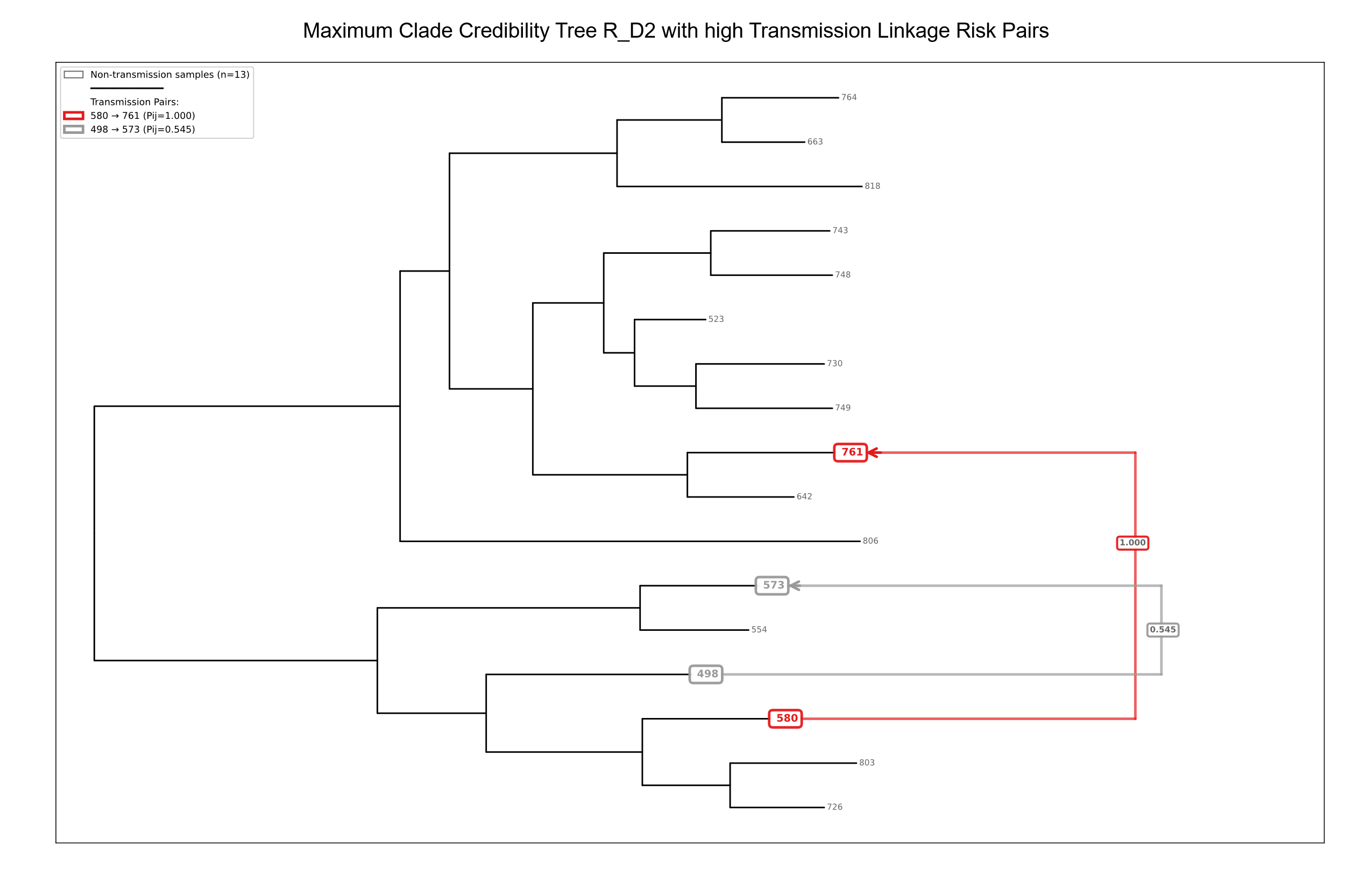


S3 Fig. Maximum Clade Credibility tree generated from cluster R (23) identified from the DENV2 samples of the Colombo Dengue Study highlighted with high transmission linkage risk pairs. The specific high probability linkages are marked by coloured arrows, and the transmission linkage risk is shown within each arrow.
