# Supplementary material for: DENcode: A model for haplotype-informed transmission probability of dengue virus
Source: PLoS Comput Biol. 2026 May 20;22(5):e1014316. doi: 10.1371/journal.pcbi.1014316 (PMC13211310; doi:10.1371/journal.pcbi.1014316)
Supplement: S1 Table — (DOCX) [file pcbi.1014316.s004.docx]

**S1 Table. Simulated directionality with the date of onset of fever**

| Serotype | Threshold | Total Edges | Edges With Dates | Concordant Edges | Concordant Percentage | Median Time Lag  (Days) |
| --- | --- | --- | --- | --- | --- | --- |
| D1 | ≥0.5 | 5 | 2 | 2 | 100 | 295.16 |
| D1 | ≥0.1 | 6 | 2 | 2 | 100 | 295.16 |
| D2 | ≥0.5 | 25 | 25 | 25 | 100 | 465.71 |
| D2 | ≥0.1 | 87 | 77 | 77 | 100 | 437.05 |
| D3 | ≥0.5 | 19 | 15 | 15 | 100 | 43.29 |
| D3 | ≥0.1 | 41 | 29 | 29 | 100 | 99.88 |
