# Supplementary material for: DENcode: A model for haplotype-informed transmission probability of dengue virus
Source: PLoS Comput Biol. 2026 May 20;22(5):e1014316. doi: 10.1371/journal.pcbi.1014316 (PMC13211310; doi:10.1371/journal.pcbi.1014316)
Supplement: S2 Table — (DOCX) [file pcbi.1014316.s005.docx]

**S2 Table. Summary of statistical comparison of transmission pairs and non-transmission branches in the two-cluster specific MCC BEAST trees.**

| Tree | Serotype | Samples | Transmission Pairs | Mean (SD)  Transmission Distance | Mean (SD)  Non-Transmission Pair Distance | Mann Whitney U test P value | Significance |
| --- | --- | --- | --- | --- | --- | --- | --- |
| R_D2 | D2 | 17 | 2 | 3.255 (1.112) | 3.084 (1.295) | 0.568 | No |
| T_D3 | D3 | 37 | 19 | 1.663 (0.436) | 1.648 (0.673) | 0.554 | No |
