# Supplementary material for: DENcode: A model for haplotype-informed transmission probability of dengue virus
Source: PLoS Comput Biol. 2026 May 20;22(5):e1014316. doi: 10.1371/journal.pcbi.1014316 (PMC13211310; doi:10.1371/journal.pcbi.1014316)
Supplement: Attachment — Submitted filename: Response to Reviewers PLOS.docx [file pcbi.1014316.s008.docx]

**Response to Reviewers**

**Manuscript title:** *DENcode: A model for haplotype‑informed transmission probability of dengue virus*
**Journal:** *PLOS Computational Biology*

We thank the Academic Editor and Reviewers for their careful evaluation of our manuscript and their constructive comments. We have revised the manuscript thoroughly to address all editorial requirements and reviewer comments. All changes are incorporated in the revised manuscript, and the added text has been highlighted. Below, we respond point‑by‑point to each comment and quote verbatim the exact text added or modified in the manuscript.

**Editorial and Journal Requirements**

| **Editorial / Journal Comment (verbatim)** | **Author Response** | **Changes Made (verbatim text added to manuscript)** |
| --- | --- | --- |
| “We do not publish any copyright or trademark symbols that usually accompany proprietary names, e.g. ©, ®, or TM.” | All copyright and trademark symbols have been removed throughout the manuscript. | All instances of copyright and trademark symbols (including ©, ®, and TM) were removed from the manuscript. |
| “We notice that your supplementary Figures and Tables are included in the manuscript file. Please remove them and upload them with the file type ‘Supporting Information’. Please ensure that each Supporting Information file has a legend listed in the manuscript after the references list.” | All supplementary figures, tables, and supplementary methods have been removed from the main manuscript and provided as a separate Supporting Information file in accordance with PLOS guidelines. | Supplementary figures, supplementary tables, and supplementary methods were removed from the main manuscript and provided in a separate file labelled “Supporting Information”. |
| “Figures 1 and S1: Please confirm whether you drew the images / clip‑art within the figure panels by hand. If you did not draw the images, please provide a link to the source and their license / terms of use.” | Figures 1 and S1 were created using BioRender.com. A BioRender publication license has been obtained, and the required attribution has been added to the figure legends. | The following text was added verbatim to the legends of Figures 1 and S1:  “Created in BioRender. Stone, H. (2026) <https://BioRender.com/ff2w68r>”  “Created in BioRender. Stone, H. (2026) https://BioRender.com/urvslx0” |
| “Figure 3: Please provide written permission from the copyright holder or replace with an open‑access alternative.” | Figure 3 was adapted from a previously published article by the authors (*Maduranga et al., Virology, 2026*). The figure legend has been updated to explicitly state this, ensuring compliance with CC BY 4.0 requirements. | The Figure 3 legend was revised verbatim to:  “Fig 3. Outline of sample selection for haplotype level analysis. Adapted from Maduranga et al., *Virology*, 2026 (26).” |
| “Figures 3 and 5: Please provide a direct link to the base layer of the map and ensure the license is compatible with our CC BY 4.0 license.” | Figure 5 uses an openly available basemap from geoBoundaries under the Creative Commons Attribution 4.0 International license, and this information is explicitly stated in the legend. | The following text was added verbatim to the Figure 5 legend:  “Administrative boundaries (Sri Lanka, ADM2) were obtained from geoBoundaries (geoBoundaries‑LKA‑ADM2) and used under the Creative Commons Attribution 4.0 International (CC BY 4.0) license.” |
| “Please amend your detailed Financial Disclosure statement. It must contain the exact wording you wish to be published and state the role of the funders.” | The Financial Disclosure statement has been revised to include full sentences, funding agencies, grant numbers, author initials, and an explicit statement of funder role. | The Financial Disclosure statement now reads verbatim:  “This work was supported by the University of Colombo, Sri Lanka (grant no. AP/3/2/2017/CG/25 to DF) and the National Health and Medical Research Council, Australia (Investigator grant no. 1173666 to CR). The funders had no role in study design, data collection and analysis, decision to publish, or preparation of the manuscript.” |

**Reviewer #1**

| **Reviewer #1 Comment (verbatim)** | **Author Response** | **Changes Made (verbatim text added to manuscript)** |
| --- | --- | --- |
| “Lines 381–418: The mathematical expressions are clearly defined, but the accompanying text would benefit from further elaboration. In particular, the background motivation for each formula and the logical relationships between successive equations are not always sufficiently explicit.” | We thank the reviewer for this helpful suggestion. We expanded the explanatory text surrounding the mathematical formulation to clarify model motivation and logical structure. | The following text was added verbatim to the Model Formulation section:  “A probabilistic pairwise framework is used because dengue transmission is only partially observed, with mosquito‑mediated spread and incomplete sampling making deterministic reconstruction of transmission pairs unreliable.”  “The resulting probability is defined relative to all candidate recipients for a given source case and is therefore interpreted as a comparative measure rather than an absolute probability of direct transmission.”  “The model integrates epidemiological feasibility and genetic consistency into a single transmission score for each candidate pair. The epidemiological kernel K_ij constrains transmission to pairs that are compatible in time, space, and vector biology, while the exponential term maps genetic divergence to a continuous penalty, reducing support for links inconsistent with recent shared ancestry. These components are combined multiplicatively so that neither epidemiological plausibility nor genetic similarity alone is sufficient to produce high transmission support.  The resulting expression defines an unnormalized score for each potential transmission pair, which is then normalised across all candidate recipients j^'≠i to obtain a conditional probability distribution. Consequently, P_ij represents the relative support for transmission from i to j among all feasible recipients of case i, rather than an absolute probability of direct infection. The epidemiological and genetic components of the model are defined in detail in the following sections.” |
| “The sample size for DENV1 is substantially smaller than for the other serotypes, and comparisons of network properties and centrality measures are therefore likely to be influenced by sample size effects. In addition, the rationale for selecting probability thresholds of 0.1 and 0.5 is not explicitly justified.” | We clarified serotype‑specific interpretation and justified probability thresholds. | The following text was added verbatim to the Network analysis section:  “Transmission networks were examined at probability thresholds of ≥0.1 and ≥0.5 to contrast moderate‑ and high‑confidence transmission linkages. The lower threshold (≥0.1) captures a broader set of epidemiologically plausible connections and provides insight into overall network coverage and structure, whereas the higher threshold (≥0.5) restricts attention to a smaller set of highly supported links, highlighting the most confident candidate transmission events Examining both thresholds allows assessment of network stability and core structure across different confidence levels, without reliance on a single arbitrary cutoff.”  The following clarification was added verbatim to the Discussion:  “Interpretation of network properties was performed primarily within individual serotypes rather than through direct quantitative comparison across serotypes. In particular, the DENV1 network was derived from a substantially smaller number of cases and likely represents background endemic transmission rather than an outbreak‑driven network.” |
| “The intersection analysis clearly demonstrates information loss when using consensus sequences; however, the extent to which this conclusion generalises to larger sample sizes or different epidemiological settings is not fully discussed.” | We expanded the Discussion to clarify scope and context dependence. | The following text was added verbatim to the Discussion:  “If haplotype‑level data is unavailable, the network connectivity at the consensus level may still improve with a larger sample size, but this aspect could not be tested with the relatively modest number of DENV2 and DENV3 sequences available to us. In addition, the degree of information loss observed with consensus‑based inference is likely to depend on the underlying epidemiological context and background viral diversity, and may vary across endemic and outbreak settings” |

**Reviewer #2**

| **Reviewer #2 Comment (verbatim)** | **Author Response** | **Changes Made (verbatim text added to manuscript)** |
| --- | --- | --- |
| “The internal validation sounds to me more as a sensitivity analysis… for internal validation to what extent the results rely on the exact signals in the input data.” | We agree and clarify the distinction between robustness assessment and external validation. | The following text was added verbatim to the Model Validation and Sensitivity Analyses section:  “These analyses quantify how sensitive inferred transmission probabilities are to specific modelling assumptions, rather than serving as validation against a known transmission ground truth.”  In addition, terminology throughout the manuscript was revised to avoid use of “internal validation” and instead emphasise robustness and sensitivity analyses. |
| “As part of the sensitivity analysis, the authors randomise the genetic distance information… Why not randomise the epidemiological information as well?” | We clarified the biological rationale for flattening rather than randomising epidemiological inputs. | The following text was added verbatim to the Sensitivity Analyses section:  “Randomisation was applied to the genetic component to assess sensitivity to the alignment between sequence similarity and case pairing. In contrast, the epidemiological kernel was flattened rather than randomised, as its inputs are constrained by temporal ordering, incubation periods, and vector survival dynamics. Randomising these quantities would produce epidemiologically inconsistent transmission scenarios. Flattening the kernel removes epidemiological structure while preserving these constraints, providing a comparable assessment of its contribution to inferred transmission probabilities.” |
| “Although the authors explain what these metrics measure, they do not further discuss the implications for the transmission network.” | We expanded the Discussion to interpret epidemiological implications and limitations of network metrics. | The following text was added verbatim to the Discussion:  “Centrality measures in DENcode-derived networks provide complementary perspectives on the potential epidemiological roles of individual cases, but should be interpreted cautiously in the context of incomplete sampling. Nodes with high out-degree or PageRank disproportionately contribute to onward transmission within the inferred network and may represent candidate transmission hubs or cases linking multiple infection chains. High betweenness centrality identifies cases that lie on many shortest paths between other nodes, suggesting a potential role as bridges between otherwise weakly connected clusters, which may reflect human mobility or spatial connectivity. In contrast, high closeness centrality indicates cases that are on average genetically and epidemiologically close to many others, placing them at central positions within the network, even if they are not highly connected by direct edges. Eigenvector-based measures further highlight nodes embedded within influential regions of the network rather than those with many direct links.  Because DENcode networks represent a probabilistic subset of true transmission events conditioned on observed cases, these metrics do not identify definitive sources or super-spreaders. Instead, they provide relative indicators of importance within the sampled transmission structure and can be used to prioritise hypotheses regarding transmission dynamics, human movement patterns, or locations where targeted surveillance or vector control may be most informative. Interpretation of network measures should therefore focus on relative patterns within a serotype-specific network rather than absolute comparisons across datasets or populations.” |
| “Line 92: ‘the virus does not … within the human host’… some mutations do arise during the infection cycle. I would suggest rewording this sentence less strongly.” | The wording was softened to acknowledge limited but non‑zero within‑host evolution. | The sentence was revised verbatim to:  “Because dengue is an acute infection, the virus has limited opportunity to accumulate mutations within a single human host compared with chronic infections such as HIV or HCV, although low‑frequency within‑host variants can still arise during the course of infection.” |
| “Line 398: Is the equation for the timespan correct? … Seems like some small mistake was made.” | The description of the infection window was corrected to remove ambiguity. | The description was revised verbatim to:  “This window spans from $t_{i}+\tau+EIP\left( T_{i} \right)$ to $t_{i}+\tau+EIP(T_{i})+\Delta$, where $EIP(T_{i})$is the temperature-dependent extrinsic incubation period in the mosquito and $\Delta$ is the maximum plausible intrinsic incubation period in the human host.” |

We believe that these revisions fully address all editorial and reviewer comments and substantially improve the clarity, rigor, and interpretability of the manuscript. We thank the editors and reviewers for their constructive feedback and the opportunity to revise our work.
